# Feasibility of a yoga intervention to decrease pain in older women: a randomized controlled pilot study

**DOI:** 10.1186/s12877-020-01818-y

**Published:** 2020-10-12

**Authors:** Rebecca Seguin-Fowler, Meredith Graham, Judy Ward, Galen Eldridge, Urshila Sriram, Diane Fine

**Affiliations:** 1 Texas A&M AgriLife Research, 600 John Kimborough Boulevard, Suite 512, College Station, TX USA; 2 Texas A&M University System, 600 John Kimborough Boulevard, Suite 512, College Station, TX USA; 3 Cornell University, 413 Savage Hall, Ithaca, NY 14853 USA; 4 Fine Spirit Yoga, 104 E. Lewis St, Ithaca, NY 14850 USA

**Keywords:** Yoga, Pain, Older women, Physical function

## Abstract

**Background:**

A significant proportion of older women suffer from chronic pain, which can decrease quality of life. The objective of this pilot randomized study was to evaluate the feasibility of a flow-restorative yoga intervention designed to decrease pain and related outcomes among women aged 60 or older.

**Methods:**

Flow-restorative yoga classes were held twice weekly for 1 hour and led by a certified yoga instructor. Participants randomized to the intervention group attended the yoga classes for 12 weeks and received supplemental materials for at-home practice. Those randomized to the control group were asked to maintain their normal daily routine. Feasibility was evaluated using recruitment and retention rates, class and home practice adherence rates, and participant satisfaction surveys. Outcome measures (self-reported pain, inflammatory markers, functional fitness, quality of life, resilience, and self-reported physical activity) were assessed at baseline and post-intervention. Paired t-tests or Wilcoxon signed-rank tests were used to examine changes in outcome measures within treatment groups.

**Results:**

Thirty-eight participants were recruited and randomized. Participants were primarily white, college-educated, and higher functioning, despite experiencing various forms of chronic pain. Attendance and retention rates were high (91 and 97%, respectively) and the majority of participants were satisfied with the yoga program (89%) and would recommend it to others (87%). Intervention participants also experienced reductions in pain interference and improvements in energy and social functioning.

**Conclusions:**

This pilot study provides essential data to inform a full scale randomized trial of flow-restorative yoga for older women with chronic pain. Future studies should emphasize strategies to recruit a more diverse study population, particularly older women at higher risk of disability and functional decline.

**Trial registration:**

Clinicaltrials.gov, NCT03790098. Registered 31 December 2018 – Retrospectively registered

## Background

Chronic pain is a complex, multifactorial condition that negatively impacts older adults’ physical and mental functioning, and quality of life [1]. According to the Centers for Disease Control and Prevention, about 30% of older adults in the United States experience some form of chronic pain [2]. Evidence also suggests that older women are more likely to experience chronic pain than older men [3–5]. For example, a nationally representative study of community-dwelling older adults found that 58% of women experienced bothersome pain in the last month compared with 47% of men [3].

Chronic pain substantially affects older adults’ mood, daily activities, mobility, and physical inactivity and has been associated with an increased risk of frailty [1, 3, 6–11] and disability. The estimated annual cost of chronic pain in the United States, including direct medical costs and lost productivity, is between $560 and $635 billion, further emphasizing the need for therapeutic intervention approaches [1].

Mind-body interventions, such as yoga, have been recommended for the management of chronic pain conditions [12, 13]. By targeting both physical and psychosocial factors, these approaches may be more effective than physical therapy or pharmacological treatment alone [14].

Yoga is an ancient spiritual practice that originated in India and integrates physical postures (asanas) with breathing and meditation techniques [15]. Yoga practice has been found to improve physical function, psychological well-being, health related quality of life, muscle strength, and balance in older adults [16]. Systematic reviews examining the effects of yoga on chronic pain have shown mixed but primarily beneficial results. Two reviews found limited evidence for the effectiveness of yoga on low back pain or rheumatoid arthritis [17, 18]. However, several reviews found moderate to strong evidence that yoga interventions can reduce symptoms associated with chronic back pain, neck pain, headaches/migraines, and fibromyalgia [12, 19–21].

Chronic pain is often experienced with inflammatory disorders (e.g. arthritis, irritable bowel disease) and inflammation has been linked to other chronic pain conditions, such as low back pain [22]. Existing studies have highlighted the association between yoga practice and reduced inflammation [23, 24]; however, only one study to date has focused on participants with chronic pain [22]. In that 12-week non-randomized study, TNF-alpha (pro-inflammatory marker) remained unchanged in the intervention group but increased significantly in the control group. No significant changes in other inflammatory markers were observed in either group.

Despite growing evidence on the potential benefits of yoga practice, few studies have focused on elderly populations with chronic pain conditions [25]. Two pilot randomized trials found that yoga reduced pain severity and pain interference among older adults with osteoarthritis [26, 27]. However, a larger randomized study found no effect of yoga on pain intensity among older adults with chronic lower back pain [28]. To date, no studies have targeted older women with any type of chronic pain or examined changes in inflammatory markers. Future randomized interventions are needed to assess the effectiveness of yoga in reducing pain symptoms and inflammation among this vulnerable population.

To address these gaps, we developed a flow-restorative yoga intervention for women age 60 or older with chronic pain. Flow yoga refers to a sequence of linked poses that are synchronized with the breath. By emphasizing stretching and strengthening through continuous movement, flow yoga sequences can help improve flexibility and strength to reduce pain symptoms. Restorative yoga consists of gentle poses, supported by props, with an emphasis on breathing and relaxation. Unlike other forms of yoga, restorative yoga promotes relaxation of muscles through passive stretching to provide pain relief [29]. The objectives of this pilot study were to evaluate the feasibility and preliminary effectiveness of flow-restorative yoga for older women to inform a fully powered randomized controlled trial.

## Methods

### Design

This study was a feasibility trial with a two-arm, parallel group, randomized-controlled design that was conducted from November 2018 to May 2019. Intervention participants received a 12-week flow-restorative yoga intervention consisting of group-based classes and take-home materials. Control participants did not receive any intervention components but were offered take-home materials after the study ended.

Participants were stratified by age (≤ 69 years or > 69 years) and C-reactive protein (CRP) level (< 2 mg/L or ≥ 2 mg/L) and randomly allocated to either intervention or control group in a 1:1 ratio. Randomization sequences were generated in Excel and implemented by an independent statistician who was unaware of group assignments and not involved in data collection.

All intervention and control group participants completed data collection procedures upon enrollment into the study (baseline) and after the 12-week intervention period (outcome). Baseline data collection took place from November 2018 to December 2018 and outcome data collection took place from April 2019 to May 2019. Participants were compensated $25 for completing all assessments at each timepoint. Study procedures and materials were approved by Cornell University’s Institutional Review Board (IRB) (Protocol # 1809008271) and Texas A&M University’s IRB (Protocol # IRB2020-0727 M).

### Sample

Participants were recruited from a micropolitan community (population approximately 30,000) in Upstate New York between November 2018 and December 2018. Eligible participants were women aged 60 years or older with self-reported chronic pain, as indicated by an average pain severity score of 3 or higher on the Brief Pain Inventory [30].

Women were excluded if they were currently practicing yoga more than four times per month, cognitively impaired, or unable/unwilling to obtain physician authorization. Those with a systolic blood pressure > 160 mmHg, diastolic blood pressure > 100 mmHg, or a resting heart rate < 55 or > 110 beats per minute were also ineligible, due to potential health risks from participating in a modestly supervised exercise intervention. Lastly, women were excluded if they were unable to get up and down from the floor (required to transition between yoga poses) or if they were unable to climb one flight of stairs (required to access the yoga studio).

Recruitment methods included flyers and emails distributed through academic listservs, county agencies, community organizations, senior housing sites, healthcare clinics, and local stores. Women who expressed interest in the study were screened via telephone after providing verbal consent. If eligible for the study, participants were required to obtain signed permission from a healthcare provider and provide written informed consent.

The target sample size of 40 participants was based on an average yoga class size of 20 and studio capacity. This feasibility trial was not designed to detect differences in outcome measures between intervention and control groups. Rather, data are being used to inform sample size calculations for a fully powered randomized controlled trial.

### Intervention

The yoga intervention integrated elements of flow and restorative yoga practices and was designed specifically for older women with chronic pain. Intervention classes were held in a local yoga studio and led by a certified yoga instructor with Cardiopulmonary Resuscitation (CPR) certification and 25 years of teaching experience in restorative yoga, breathwork and meditation practices. The yoga intervention was co-developed by the yoga instructor and the principal investigator who had extensive experience developing physical activity programs for older women and was a Certified Strength and Conditioning Specialist (CSCS) and trained PranaVayu yoga instructor.

Classes took place twice weekly during the 12-week intervention period (January 2019 – April 2019), with two make-up sessions available. Each hour-long class consisted of breathwork (5 min), standing and seated flow postures (40 min), and restorative postures (15 min). Participants with mobility constraints were offered pose modifications and supportive props, including blocks, blankets, and chairs. The postures and modifications were common and similar to other restorative, flow, and Hatha yoga postures. Examples of modifications included reducing the full range of motion if full extension was impossible or caused pain, or using a chair, towel, and/or bolster to assist with proper and comfortable body positioning.

Additional file 1 depicts the general sequence of postures included in each class. The full intervention manual, including pose descriptions, timing, and modifications, can be made available upon request to the primary author. Intervention participants also received a supplemental booklet and video of class poses to take home and were encouraged to practice at least two additional days per week. Control group participants were asked not to begin yoga classes and all participants were asked not to begin any new exercise programs during the 12-week intervention period.

### Measures

Demographic (age, race, ethnicity, education, income), psychosocial, and health-related measures were assessed using paper-based or online Qualtrics surveys. Functional fitness measures were assessed by trained research personnel (blinded to intervention assignment) during in-person data collection visits.

Primary outcome measures included self-reported pain and inflammatory markers (CRP and cytokines IL-2, IL-1β, TNF-α, IFN-γ, IL-6, IL-4, and IL-10). Pain severity and interference with daily activities were measured with the Brief Pain Inventory (BPI) [30]. The BPI is a reliable, valid tool that has been recommended for use in all clinical trials focused on chronic pain [31]. Pain severity consisted of four items that assessed worst pain, least pain, average pain, and current pain. Pain interference was calculated by averaging seven items that asked how much pain interfered with daily activities (general activity, walking, work, mood, enjoyment of life, relations with others, and sleep). All items were rated on a scale from 0 to 10.

To measure inflammatory markers, venous blood draws were performed and samples were assayed using the Siemens Immulite 2000 immunoassasy system (CRP) or Luminex Magpix multiplex immunoassay system (inflammatory cytokines).

Secondary outcome measures included health-related quality of life, functional fitness, resilience, and physical activity. Health-related quality of life was measured using the RAND 36-Item Short Form Survey, which consists of eight subscales to assess various aspects of physical and mental health [32]. Functional fitness was assessed using the Senior Fitness Test, which is comprised of six tests to measure strength, endurance, flexibility, and agility [33]. Resilience was assessed using the Brief Resilience Scale and physical activity frequency and duration were assessed using the Community Healthy Activities Model Program for Seniors (CHAMPS) Activities Questionnaire for Older Adults [34, 35].

Feasibility measures included demand, safety, contamination, and acceptability [36]. Demand (use of and estimated need for the intervention) was determined by recruitment, retention, and yoga adherence rates. Information on recruitment and retention were collected by study personnel. Adherence was assessed using class attendance records and participant-reported frequency of home practice.

Safety was assessed through reporting of adverse events during and outside of yoga classes. Adverse events were defined as any unexpected injuries or symptoms including falls, fractures, sprains, muscle strains, and joint pain. Adverse events were monitored by the yoga instructor during each class and any events were reported back to the research team within 24 h. Participants also completed a health history form at baseline and post-intervention to monitor adverse events occurring outside of class sessions.

Contamination was assessed by asking participants to report on yoga and other exercise practices at baseline and post-intervention. Other exercise practices included stretching or flexibility exercises (not including yoga) and general conditioning (e.g. light calisthenics or chair exercises).

Lastly, acceptability was evaluated through participant satisfaction surveys administered post-intervention. Survey questions asked about class progression and variation, perceived difficulty, satisfaction with program components, and benefits of participation. Questions related to progression, variation, and difficulty were assessed using 10-point scales while questions related to satisfaction and participation benefits were assessed using 5-point scales. Participants were also asked open-ended questions about program perceptions and recommendations for improvement.

### Statistical analysis

Quantitative data were analyzed using IBM SPSS Statistics for Macintosh (version 25.0, IBM Corp., Armonk, NY; 2017). Baseline characteristics (demographics and outcome measures) and feasibility measures were summarized using means for continuous variables and frequencies for categorical variables. T tests (continuous), Mann-Whitney U tests (continuous, non-normally distributed), or Fisher’s exact tests (categorical) were used to compare differences in baseline characteristics between groups. This pilot study was not powered to examine between group differences in outcome measures. However, pre-post changes in outcome measures within groups were assessed using paired t tests (normally distributed) or Wilcoxon signed-rank tests (non-normally distributed).

Open-ended responses were qualitatively coded using Microsoft Excel 2014. Responses to each survey question were coded separately and similar codes were subsequently grouped together. Codes were categorized to reflect cross-cutting themes: positive aspects of the program, negative aspects of the program, participation benefits, participation challenges, and recommendations for improvement. Coding decisions were reviewed by two members of the research team and iteratively revised. Final codes were enumerated to compare the frequency of participant responses. All analyses were conducted in 2019–2020.

## Results

**Fig. 1:**
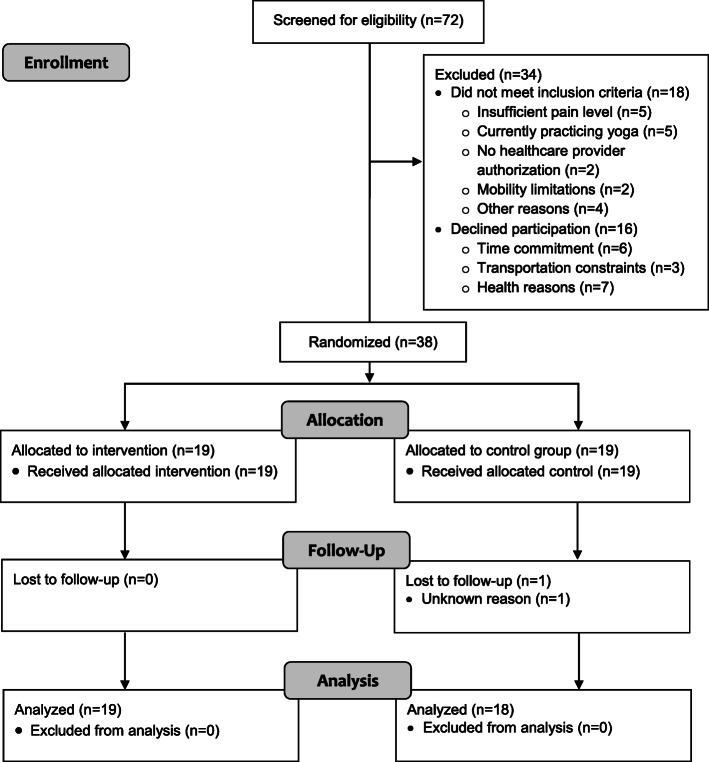
CONSORT Flow Diagram

A total of 72 women were screened for eligibility and 54 women (75%) were deemed eligible for participation (Fig. 1). Primary reasons for ineligibility were insufficient pain severity or current yoga practice of 5 or more times per month. Only two individuals were excluded due to mobility limitations (i.e. unable to get up and down from the floor).

Of those screened, 38 women enrolled in the study (52% recruitment rate) and were randomized to yoga intervention (*n* = 19) or control (*n* = 19). Common reasons for declining participation among eligible individuals were health constraints (e.g. recent surgery) and time commitments (e.g. unable to take time off work). Participants who enrolled in the study reported a variety of pain conditions, including back pain, arthritis, fibromyalgia, chronic headaches, and joint pain (e.g. hip, shoulder, and knee). Average pain severity ranged from 3 to 7 with a mean score of 4.3 among all participants. Thirty-seven participants attended the outcome data collection visit (97% retention rate) and all intervention participants completed the satisfaction survey.

Participant baseline characteristics are presented in Table 1. Intervention participants reported higher pain interference as compared to control participants (diff: 1.5, *p* = 0.054). No significant differences in demographic characteristics or other outcome measures were observed between groups.

**Table 1:** Participant Baseline Characteristics by Group (*N* = 38)

Characteristic	Intervention (n = 19)	Control (n = 19)	***P***
**Age**	66 (7.3)	65 (4.0)	0.61
≤ 69 years	16 (84.2)	16 (84.2)	–
> 69 years	3 (15.8)	3 (15.8)	–
**Race/ethnicity (n,%)**			
Non-Hispanic white	16 (84.2)	17 (89.4)	1.00
Hispanic	1 (5.3)	0 (0.0)	
Native Hawaiian/Pacific Islander	0 (0.0)	1 (5.3)	
Prefer not to answer	2 (10.5)	1 (5.3)	
**Education (n,%)**			
Some college	3 (15.8)	2 (10.5)	0.65
College graduate	4 (21.1)	7 (36.8)	
Post-grad/professional	12 (63.2)	10 (52.6)	
**Marital Status (n,%)**			
Married or cohabiting	11 (57.9)	11 (61.1)	1.00
Living alone	8 (42.1)	7 (38.9)	
**Employment (n,%)**			
Working	8 (42)	9 (47.4)	1.00
Not working	11 (58)	10 (52.6)	
**Income (n,%)**			
Less than $50,000	5 (29.4)	9 (56.3)	0.17
$50,000+	12 (70.6)	7 (43.8)	
**Currently Practicing Yoga**			
Yes	2 (10.5)	1 (5.2)	**–**
Frequency (times/week)	1	1	**–**
**Pain Symptoms (BPI)**			
Pain interference (range: 0–10)	4.5 (2.1)	3.1 (2.0)	0.054
Worst pain (range: 0–10)	5.8 (1.6)	5.3 (1.4)	0.29
Least pain (range: 0–10)	2.6 (1.5)	2.0 (1.6)	0.22
Average pain (range: 0–10)	4.2 (1.4)	3.7 (1.5)	0.31
Current pain (range: 0–10)	3.0 (1.8)	2.4 (1.6)	0.42
**C-Reactive Protein** (mg/L)	3.5 (3.8)	2.8 (3.8)	0.34
**Quality of Life (SF-36)**			
Physical functioning (range: 0–100)	56.4 (20.9)	65.8 (23.1)	0.21
Role limitations (physical) (range: 0–100)	22.2 (35.2)	30.3 (38.7)	0.47
Role limitations (emotional) (range: 0–100)	55.6 (44.3)	61.4 (38.9)	0.71
Energy/fatigue (range: 0–100)	40.8 (20.9)	45.0 (16.8)	0.50
Well-being (range: 0–100)	62.1 (22.0)	73.6 (11.3)	0.11
Social functioning (range: 0–100)	58.6 (26.0)	71.7 (18.6)	0.12
Pain (range: 0–100)	45.9 (17.9)	51.7 (11.4)	0.34
General health (range: 0–100)	53.4 (20.2)	60.0 (22.5)	0.35
**Functional Fitness (SFT)**			
8-ft up-and-go (s)	5.9 (1.3)	5.6 (0.9)	0.54
30-s arm curl (reps)	22.4 (5.9)	19.7 (3.8)	0.21
30-s chair stand (reps)	16.6 (5.9)	15.0 (3.6)	0.58
Back scratch (cm)	10.4 (10.0)	6.4 (12.4)	0.28
Chair sit-and-reach (cm)	−3.1 (10.6)	0.03 (7.3)	0.30
2-min step test (steps)	98 (26.3)	90.3 (23.6)	0.34
**Resilience (BRS)**	3.2 (0.8)	3.7 (0.8)	0.06
**Physical Activity (CHAMPS)**			
Total PA frequency (times/wk)	19.0 (17.5)	20.6 (9.1)	0.28
Moderate PA frequency (times/wk)	9.0 (8.1)	9.6 (5.8)	0.45
Total PA duration (hr/wk)	14.8 (10.5)	16.4 (10.0)	0.56
Moderate PA duration (hr/wk)	7.0 (5.8)	7.2 (6.2)	0.84

**Note:** Values are expressed as means (SD) unless otherwise specified

**Abbreviations:** *BPI* Brief Pain Inventory, *SF-36* 36-Item Short Form Survey, *SFT* Senior Fitness Test, *BRS* Brief Resilience Scale, *CHAMPS* Community Healthy Activities Model Program for Seniors, *PA* Physical activity

Among those who were screened for eligibility, the most common recruitment channels were academic listservs (*n* = 23, 32%), senior centers/housing sites (*n* = 14, 19%), and referrals from friends (*n* = 10, 14%). Similarly, most participants who enrolled in the study were recruited via academic listservs (*n* = 13, 34%), senior centers/housing sites (*n* = 6, 16%), or referrals (n = 6, 16%).

On average, participants in the intervention group attended 91% of yoga classes (range: 54 to 100%). Of the 19 intervention participants, 18 attended > 80% of classes. Eighty-eight percent of participants used the yoga booklet at least once when practicing at home and 39% used it at least once per week. Sixty-six percent used the yoga video at least once and 17% used it at least once per week, with most participants preferring the DVD format over online streaming or USB. No adverse events or injuries related to yoga practice were observed during class or reported by participants.

Only one participant in the control group reported practicing yoga at baseline and continued this routine throughout the study period. No other control group participants reported practicing yoga at baseline or post-intervention. Two intervention participants and five control participants reported starting another exercise practice during the study. Of these, one intervention participant and three control participants started stretching/flexibility exercises, one intervention participant started general conditioning, and two control participants started both types of exercise.

At the beginning of the program, 28% of women found the class difficulty just about right or too easy. By the end of 12 weeks, 83% found the class difficulty just about right or too easy. Overall, participants reported that classes either progressed at the right rate (61%) or slower than expected (39%). Conversely, 61% of participants felt that class sequences did not have enough variation, while 39% were satisfied with the degree of variation.

The majority of intervention participants were satisfied with the yoga program (89%) and indicated that they would recommend the program to other women (87%). Most women found the instructor very easy to follow (83%) and appreciated the availability of props and pose modifications (61 and 78%, respectively) (Table 2). The majority of participants reported some improvements in strength, physical activity, energy level, range of movement, pain symptoms, mood, and overall health (range: 50 to 72%) (Table 2).

**Table 2:** Intervention Participant Class Experience Survey, Quantitative Results (*N* = 18)

	Very Much n (%)	Somewhat/Mostly n (%)	A little/Not at all n (%)
**Program Satisfaction**			
Overall satisfaction	9 (50.0)	7 (38.9)	2 (11.1)
Class usefulness	10 (55.6)	6 (33.3)	2 (11.1)
Easy to follow	15 (83.3)	3 (16.7)	0 (0.0)
Prop usefulness	11 (61.1)	6 (33.3)	1 (5.6)
Pose modifications	14 (77.8)	3 (16.7)	1 (5.6)
**Participation Benefits**			
Increased strength	4 (22.2)	10 (55.6)	4 (22.2)
More active	1 (5.6)	13 (72.2)	4 (22.2)
Increased energy	1 (5.6)	11 (61.1)	6 (33.3)
Better sleep quality	1 (5.6)	9 (50.0)	8 (44.4)
Improved range of movement	1 (5.6)	12 (66.7)	5 (27.8)
Improved pain symptoms	1 (5.6)	9 (50.0)	7 (38.9)
Improved mood	3 (16.7)	11 (61.1)	4 (22.2)
Improved overall health	1 (5.6)	11 (61.1)	6 (33.3)

Table 3 presents key themes that emerged from participants’ open-ended responses, along with response frequencies and supporting quotes. Several participants acknowledged the benefits of practicing a consistent routine (33%) and enjoyed developing their yoga practice (28%).

**Table 3:** Intervention Participant Class Experience Survey, Qualitative Results (*N* = 18)

Theme	Subtheme	Frequency n (%)	Supporting Quotes
Positive Aspects	*Developing skills*	5 (28)	“Learning new yoga poses that are easy to use every day in daily life (e.g. work stretch break).”
	*Consistent routine*	6 (33)	“With repetitiveness, I was able to practice and get better with the correct way to hold postures.”
	*Restorative*	10 (56)	“Enjoyed legs up the wall to reverse blood flow.”; “I appreciated the chance to disconnect and relax.”
	*Supportive instructor*	6 (33)	“[Instructor] was gently supportive and encouraged us to do what felt right for our body.”
	*Useful home materials*	5 (28)	“I thought the materials were excellent and I appreciated having both formats.”
Negative Aspects	*Progress plateau*	2 (11)	“I did hit a plateau so did not see further improvement after halfway.”
	*Class sequence*	5 (28)	“Restorative poses were too lengthy.”
Participation Benefits	*Gaining confidence*	5 (28)	“Once I figured out the pose transitions, I began to progress and feel I could really do this.”
	*Improving fitness*	8 (44)	“I built stamina quickly. Yoga definitely helped with building strength as well.”
	*Relieving pain*	2 (11)	“I had severe hip pain daily before the yoga practice. Now I have it infrequently.”
Participation Challenges	*Physical limitations*	8 (44)	“I had one flare of inflammation, which limited my ability to participate for a few days.”
	*Learning curve*	5 (28)	“It was a very new experience for me, so rather difficult until I developed some motor memory.”
	*No pain relief*	5 (28)	“I do not feel my back pain has lessened due to yoga.”
Recommendations	*Additional poses*	10 (56)	“Near the end, I would have appreciated the introduction of additional poses to augment what we learned.”
	*Modify class sequence*	7 (39)	“More restorative poses, less flow yoga, more time for pause at the end of each pose.”
	*Modify class schedule*	5 (28)	“It would be nice to have evening classes for those of us who work.”
	*Studio location*	5 (28)	“[Did not enjoy] going up the stairs, would have classes on the first floor.”
	*Improve home materials*	6 (33)	“I would have liked two DVDs, one instructional and one with the “real time” practice flow.”

“With repetitiveness, I was able to practice and get better with the correct way to hold postures.”

More than half of participants appreciated the restorative nature of yoga sessions, which helped them to relax both mentally and physically. Additionally, several women (33%) credited their progress to the guidance and encouragement provided by the class instructor.

“[Instructor] was gently supportive and encouraged us to do what felt right for our body.”

A few challenging aspects of the yoga program were also discussed. Some participants (28%) expressed dissatisfaction with the duration of restorative poses or specific flow poses that were too challenging. Others (11%) described hitting a “progress plateau” halfway through the program, with no further improvements in strength and stamina.

Benefits of participation included gaining confidence, improving fitness, and relieving pain. Several women (28%) reported increasing comfort and familiarity with class poses as the program progressed, which boosted their self-confidence. About half of participants described improvements in physical fitness (e.g. strength) and/or mental well-being as a result of consistent yoga practice, while a few (11%) also experienced reductions in pain symptoms.

“I had severe hip pain daily before the yoga practice. Now I have it infrequently.”

Physical pain or fear of injury limited many participants (44%) from fully engaging in yoga sessions, especially earlier on in the program. Additional challenges to participation included the steep learning curve and not experiencing pain relief. Several women (28%) reported difficulties due to unfamiliarity with poses or breathing patterns.

“It was a very new experience for me, so rather difficult until I developed some motor memory.”

Participants offered multiple recommendations for improvement, including modifications to the class sequence and schedule. More than half of women expressed their desire for more challenging poses by the end of the program. Others (39%) suggested varying the routine or modifying the duration of poses.

“More restorative poses, less flow yoga, more time for pause at the end of each pose.”

Some participants (28%) recommended extending class length, offering classes more frequently, or shifting the timing of classes to accommodate work schedules. Additional recommendations included choosing a more accessible studio location (28%) and modifying the at-home materials (33%). Participants most commonly requested separate DVDs for learning the poses and moving through the full sequence.

“I would have liked two DVDs, one instructional and one with the “real time” practice flow.”

Intervention participants experienced significant reductions in pain interference (*P* = 0.006) (Table 4). Reductions in severity of worst pain and CRP levels were also observed; however, these changes were not significant. The majority of participants had cytokine levels below the detection threshold at both time points (baseline: *n* = 37 (98%), outcome: *n* = 36 (95%); average across all cytokines measured); thus, the results are not presented.

**Table 4:** Within-Group Changes in Outcome Measures among Intervention and Control Participants (*N* = 38)

	Intervention (n = 19)			Control (n = 19)		
	Baseline	Outcome	***P***	Baseline	Outcome	***P***
**Pain Symptoms (BPI)**						
Pain interference (range: 0–10)	4.4 (2.1)	3.2 (2.2)	**0.006**	3.1 (2.0)	2.4 (2.4)	0.08
Worst pain (range: 0–10)	5.8 (1.6)	5.0 (1.7)	0.11	5.3 (1.4)	5.0 (2.0)	0.66^a^
Least pain (range: 0–10)	2.6 (1.5)	2.2 (2.2)	0.57	2.0 (1.6)	1.6 (1.9)	0.23
Average pain (range: 0–10)	4.2 (1.4)	3.9 (2.0)	0.33^a^	3.7 (1.5)	3.4 (1.8)	0.43^a^
Current pain (range: 0–10)	3.0 (1.8)	3.1 (1.9)	0.92	2.4 (1.6)	2.2 (2.0)	0.25
**C-Reactive Protein,** mg/L	3.5 (3.8)	3.0 (3.5)	0.31	2.8 (3.8)	2.2 (2.3)	0.95^a^
**Quality of Life (SF-36)**						
Physical functioning (range: 0–100)	56.4 (20.9)	60.6 (20.6)	0.22	65.8 (23.1)	64.2 (29.4)	0.68
Physical health limitations (range: 0–100)	22.2 (35.2)	36.1 (39.0)	0.32^a^	30.3 (38.7)	54.2 (41.3)	**0.02**^a^
Emotional health limitations (range: 0–100)	55.6 (44.3)	66.7 (41.2)	0.23^a^	61.4 (38.9)	81.5 (30.7)	0.13
Energy/fatigue (range: 0–100)	40.8 (20.9)	53.3 (21.3)	**0.01**	45.0 (16.8)	47.5 (18.5)	0.46
Well-being (range: 0–100)	62.1 (22.0)	70.5 (17.0)	0.16	73.6 (11.4)	76.0 (15.0)	0.67
Social functioning (range: 0–100)	58.6 (26.0)	76.4 (24.6)	**0.004**^a^	71.7 (18.6)	76.4 (23.8)	0.41
Pain (range: 0–100)	45.9 (17.9)	57.1 (18.5)	**0.02**	51.7 (11.4)	59.3 (17.5)	0.08
General health (range: 0–100)	53.4 (20.2)	58.8 (20.1)	0.24	60.0 (22.5)	64.4 (19.3)	0.42^**a**^
**Functional Fitness (SFT)**						
8-ft up-and-go (s)	5.9 (1.3)	6.1 (2.2)	0.79^a^	5.6 (0.94)	5.7 (0.86)	0.82
30-s arm curl (reps)	22.4 (5.9)	23.5 (5.5)	0.36	19.7 (3.8)	20.6 (3.6)	0.40
30-s chair stand (reps)	16.6 (5.9)	16.5 (5.9)	0.94	15.0 (3.6)	15.9 (4.1)	0.11
Back scratch (cm)	10.4 (10.0)	8.9 (11)	0.36	6.4 (12)	3.6 (6.4)	0.27^a^
Chair sit-and-reach (cm)	−3.1 (10.6)	−2.7 (13)	0.83	0.03 (7.3)	0.5 (8.2)	0.77
2-min step test (steps)	98.0 (26.3)	93.4 (34)	0.43	90.3 (23.6)	93.3 (25.0)	**0.02**^a^
**Resilience (BRS)**	3.2 (0.8)	3.5 (0.88)	0.12	3.7 (0.8)	3.8 (0.7)	0.85
**Physical Activity (CHAMPS)**						
Total PA frequency (times/wk)	19.0 (17.6)	18.9 (9.4)	0.50^a^	20.6 (9.1)	23.3 (11.2)	0.30
MVPA frequency (times/wk)	9.0 (8.0)	6.7 (5.1)	0.45^a^	9.6 (5.8)	10.2 (5.4)	0.57
Total PA duration (hr/wk)	14.8 (10.5)	14.5 (7.0)	0.91^a^	16.4 (10.0)	15.1 (9.0)	0.80
MVPA duration (hr/wk)	7.0 (5.8)	5.3 (4.8)	0.27^a^	7.2 (6.2)	7.0 (5.2)	0.65

**Note:** Boldface indicates significant pre-post changes within group (*p* < 0.05). Pre-post changes were evaluated using paired *t*-tests or Wilcoxon signed-rank tests

^a^Wilcoxon signed-rank tests for non-parametric data

**Abbreviations:** *BPI* Brief Pain Inventory, *SF-36* 36-Item Short Form Survey, *SFT* Senior Fitness Test, *BRS* Brief Resilience Scale, *CHAMPS* Community Healthy Activities Model Program for Seniors, *PA* Physical activity, *MVPA* Moderate/vigorous physical activity

Improvements were observed across all quality of life (QoL) domains in the intervention group, with participants reporting improvements in energy/fatigue (*P* = 0.01), social functioning (*P* = 0.004), and pain (*P* = 0.02). Although this study was not powered to detect differences between groups, control participants reported smaller changes across most QoL domains (Table 4).

Participants in both groups improved across functional fitness measures of upper body flexibility (back scratch test) and strength (arm curl), although changes were not significant. Control group participants also experienced improvements in aerobic endurance (2-min step test, *P* = 0.02) and self-reported frequency of physical activity. No improvements in aerobic endurance (2-min step test), agility (8-ft up-and-go), or self-reported frequency and duration of physical activity were observed among intervention participants (Table 4).

## Discussion

Chronic pain imposes a significant burden upon older women’s quality of life, health, and longevity [1]. However, research on the effectiveness of therapeutic interventions for this population is sparse. The present study aimed to evaluate a flow-restorative yoga intervention designed to reduce pain and related outcomes in older women.

Strengths of this study include the randomized controlled design, comprehensive assessment of implementation feasibility, and measurement of multiple health-related outcomes. Our findings support the feasibility and potential benefits of regular yoga practice; however, larger trials are needed to confirm the effectiveness of this approach.

Using various recruitment strategies and channels, we successfully enrolled 95% of our target sample size over two months. Importantly, 95% of control participants and 100% of intervention participants were retained in the study. This was likely attributable to a combination of factors including study reminders, financial incentives, effective communication between participants and research personnel, and the group-based intervention setting [37].

Overall, participants were satisfied with the yoga program and would readily recommend it to other women in their community. Average attendance was high (91%), with the majority of participants attending 80% or more of program classes. Due to consistent adherence and the repeatability of class sequences, participants reported progressive improvements in their yoga practice. In addition, postures were easily adaptable for those with physical constraints. These findings suggest that implementing feasible and acceptable programs can help address low rates of exercise adherence among older adults [38].

Intervention participants experienced reductions in pain interference and improvements in several quality of life domains (social functioning, energy/fatigue, and pain), indicating a more favorable health state. In particular, increases in social functioning highlight the importance of group-based yoga classes among older adults. Future studies should be sufficiently powered to compare changes in outcome measures between groups and adjust for any baseline differences in these measures (e.g. differences in pain interference observed between intervention and control participants). No significant changes in pain severity, aerobic endurance, agility, or self-reported physical activity were observed among intervention participants. Lack of improvement in several outcomes may be due to the small sample size. Based on this study, the effect of yoga practice as a bridge to other types of activity remains unclear.

As mentioned, although older people are more likely to experience chronic pain, few studies examining the relationship between yoga and chronic pain have focused on elderly populations [25]. Similar to this study, one pilot randomized trial of yoga for older adults with osteoarthritis found reduced pain interference and fatigue in intervention participants [26]. A randomized pilot trial of yoga for older women with knee osteoarthritis reported quality of life as two scores: mental and physical summary scales; there were no significant effects on either of these summary scales [27]. This is in contrast to the present study where we found significant improvements for intervention participants in some quality of life measures (energy/fatigue, social functioning, and pain). Although not specific to older populations, reviews of the effects of yoga on chronic pain have also reported that yoga decreases fatigue, pain, and pain interference and improves quality of life [39–42].

Two 2018 reviews concluded there was an overall pattern of pro-inflammatory downregulation with the practice of yoga [23, 24]. As mentioned, only one yoga study to date has measured inflammatory markers in participants with chronic pain [22]. In that study, similar to the present study, there was no change in CRP levels for participants [22]. In the present study, cytokine levels were below the minimum detection thresholds. Our inability to detect cytokine levels may have resulted from using enrollment thresholds for pain severity (i.e. score of 3 or higher) that were too low to observe any associated inflammation. Alternative explanations include the enrollment of participants with various forms of pain, which may or may not have been associated with inflammatory symptoms. Future studies examining the effect of yoga on inflammation might consider focusing on participants with similar types of pain (e.g. low back pain, neck pain, osteoarthritis) and with higher pain severity.

Several limitations of this research should be considered. This study was not sufficiently powered to detect differences between groups; however, the significant effects and positive trends observed in several outcome measures provide support for larger controlled studies. We recruited a convenience sample of participants for the purposes of this pilot study. However, this resulted in a higher functioning, predominantly white, and socioeconomically advantaged study population. Future studies should recruit participants from multiple geographic areas and focus on community locations with diverse populations, such as senior centers, public housing sites, and assisted living facilities. Enrollment of individuals with lower functional status and disabling pain was likely limited by the study inclusion and exclusion criteria. For example, women were excluded if they were unable to climb a flight of stairs to access the yoga studio. Future studies should ensure that study locations are accessible for individuals with mobility or functional limitations. In addition, the average pain severity threshold (3 or higher) was likely insufficient to identify low-functioning individuals or those with severe chronic pain. Using additional measures of pain duration and frequency may help address this limitation. Fidelity monitoring was not conducted, which is an additional limitation to the study; future studies should conduct site visits to measure fidelity to the intervention protocol. No adverse events were reported post-intervention by participants or the instructor; future studies could include surveys at each class to ask about changes in pain since the last class.

## Conclusions

This study provides essential quantitative and qualitative data to inform a well-designed and fully powered randomized trial to evaluate a yoga intervention for pain and related health outcomes and behaviors in older women. Future studies should focus on inclusive recruitment strategies to assess the effectiveness of this approach among racially/ethnically diverse, socioeconomically disadvantaged, and lower functioning older women.

## Supplementary information

**Additional file 1.** Class Sequence of Yoga Postures. Pictorial representation of the sequence of yoga postures included in each class.

## Data Availability

The datasets generated and/or analyzed for the current study will be deposited in an online data portal for public use; however, this portal is not yet available. In the interim, datasets will be available from the corresponding author upon reasonable request.

## Abbreviations

BPI: Brief Pain Inventory
CHAMPS: Community Healthy Activities Model Program for Seniors
CPR: Cardiopulmonary Resuscitation
CSCS: Certified Strength and Conditioning Specialist
QoL: Quality of Life

## References

[1] Institute of Medicine (2011). Relieving pain in America: a blueprint for transforming prevention, care, education, and research.

[2] Dahlhamer J, Lucas J, Zelaya C, Nahin R, Mackey S, DeBar L (2018). Prevalence of chronic pain and high-impact chronic pain among adults — United States, 2016. MMWR Morb Mortal Wkly Rep.

[3] Patel KV, Guralnik JM, Dansie EJ, Turk DC (2013). Prevalence and impact of pain among older adults in the United States: findings from the 2011 National Health and aging trends study. Pain..

[4] Leveille SG, Zhang Y, McMullen W, Kelly-Hayes M, Felson DT (2005). Sex differences in musculoskeletal pain in older adults. Pain..

[5] Tsang A, von Korff M, Lee S, Alonso J, Karam E, Angermeyer MC (2008). Common chronic pain conditions in developed and developing countries: gender and age differences and comorbidity with depression-anxiety disorders. J Pain.

[6] Gibson SJ, Lussier D (2012). Prevalence and relevance of pain in older persons. Pain Med.

[7] Rodríguez-Sánchez I, García-Esquinas E, Mesas AE, Martín-Moreno JM, Rodríguez-Mañas L, Rodríguez-Artalejo F (2018). Frequency, intensity and localization of pain as risk factors for frailty in older adults. Age Ageing.

[8] Saraiva MD, Suzuki GS, Lin SM, de Andrade DC, Jacob-Filho W, Suemoto CK (2018). Persistent pain is a risk factor for frailty: a systematic review and meta-analysis from prospective longitudinal studies. Age Ageing.

[9] Bindawas S, Vennu V, Stubbs B (2018). Longitudinal relationship between knee pain status and incident frailty: data from the osteoarthritis initiative. Pain Med.

[10] Chiou J-H, Liu L-K, Lee W-J, Peng L-N, Chen L-K (2018). What factors mediate the inter-relationship between frailty and pain in cognitively and functionally sound older adults? A prospective longitudinal ageing cohort study in Taiwan. BMJ Open.

[11] Wade KF, Marshall A, Vanhoutte B, Wu FCW, O’Neill TW, Lee DM (2017). Does pain predict frailty in older men and women? Findings from the English longitudinal study of ageing (ELSA). J Gerontol A Biol Sci Med Sci.

[12] Büssing A, Ostermann T, Lüdtke R, Michalsen A (2012). Effects of yoga interventions on pain and pain-associated disability: a meta-analysis. J Pain.

[13] Schmid AA, Fruhauf CA, Sharp JL, van Puymbroeck M, Bair MJ, Portz JD (2019). Yoga for people with chronic pain in a community-based setting: a feasibility and pilot RCT. J Evid Based Integr Med.

[14] Lee C, Crawford C, Hickey A, Active Self-Care Therapies for Pain (PACT) Working Group (2014). Mind–body therapies for the self-management of chronic pain symptoms. Pain Med.

[15] Nayak NN, Shankar K (2004). Yoga: a therapeutic approach. Phys Med Rehabil Clin N Am.

[16] Sivaramakrishnan D, Fitzsimons C, Kelly P, Ludwig K, Mutrie N, Saunders DH (2019). The effects of yoga compared to active and inactive controls on physical function and health related quality of life in older adults - systematic review and meta-analysis of randomised controlled trials. Int J Behav Nutr Phys Act.

[17] Wieland LS, Skoetz N, Pilkington K, Vempati R, D’Adamo CR, Berman BM (2017). Yoga treatment for chronic non-specific low back pain. Cochrane Database Syst Rev.

[18] Cramer H, Lauche R, Langhorst J, Dobos G (2013). Yoga for rheumatic diseases: a systematic review. Rheumatology..

[19] Posadzki P, Ernst E, Terry R, Lee MS (2011). Is yoga effective for pain? A systematic review of randomized clinical trials. Complement Ther Med.

[20] Cramer H, Lauche R, Haller H, Dobos G (2013). A systematic review and meta-analysis of yoga for low back pain. Clin J Pain.

[21] Langhorst J, Klose P, Dobos GJ, Bernardy K, Häuser W (2013). Efficacy and safety of meditative movement therapies in fibromyalgia syndrome: a systematic review and meta-analysis of randomized controlled trials. Rheumatol Int.

[22] Cho HK, Moon W, Kim J (2015). Effects of yoga on stress and inflammatory factors in patients with chronic low back pain: a non-randomized controlled study. Eur J Integr Med.

[23] Moraes LJ, Miranda MB, Loures LF, Mainieri AG, Mármora CH (2018). A systematic review of psychoneuroimmunology-based interventions. Psychol Health Med.

[24] Falkenberg RI, Eising C, Peters ML (2018). Yoga and immune system functioning: a systematic review of randomized controlled trials. J Behav Med.

[25] Reid MC, Papaleontiou M, Ong A, Breckman R, Wethington E, Pillemer K (2008). Self-management strategies to reduce pain and improve function among older adults in community settings: a review of the evidence. Pain Med.

[26] Park J, McCaffrey R, Newman D, Liehr P, Ouslander JG (2017). A pilot randomized controlled trial of the effects of chair yoga on pain and physical function among community-dwelling older adults with lower extremity osteoarthritis. J Am Geriatr Soc.

[27] Cheung C, Wyman JF, Resnick B, Savik K (2014). Yoga for managing knee osteoarthritis in older women: a pilot randomized controlled trial. BMC Complement Altern Med.

[28] Teut M, Knilli J, Daus D, Roll S, Witt CM (2016). Qigong or yoga versus no intervention in older adults with chronic low back pain—a randomized controlled trial. J Pain.

[29] Fischer-White TG, Anderson JG, Lewis JE, Rose KM, Taylor AG (2015). Restorative yoga for symptom management in fibromyalgia: results of an 8-week intervention. J Yoga Phys Ther.

[30] Stanhope J (2016). Brief pain inventory review. Occup Med.

[31] Dworkin RH, Turk DC, Farrar JT, Haythornthwaite JA, Jensen MP, Katz NP (2005). Core outcome measures for chronic pain clinical trials: IMMPACT recommendations. Pain.

[32] Ware J, Sherbourne C (1992). The MOS 36-item short-form health survey (SF-36). I. Conceptual framework and item selection. Med Care.

[33] Rikli R, Jones C (2012). Senior fitness test manual - second edition.

[34] Smith BW, Dalen J, Wiggins K, Tooley E, Christopher P, Bernard J (2008). The brief resilience scale: assessing the ability to bounce back. Int J Behav Med.

[35] Stewart A, Mills K, King A, Haskell W, Gillis D, Ritter P (2001). CHAMPS physical activity questionnaire for older adults: outcomes for interventions. Med Sci Sports Exerc.

[36] Bowen DJ, Kreuter M, Spring B, Cofta-Woerpel L, Linnan L, Weiner D (2009). How we design feasibility studies. Am J Prev Med.

[37] Abshire M, Dinglas VD, Cajita MIA, Eakin MN, Needham DM, Himmelfarb CD (2017). Participant retention practices in longitudinal clinical research studies with high retention rates. BMC Med Res Methodol.

[38] Findorff MJ, Wyman JF, Gross CR (2009). Predictors of long-term exercise adherence in a community-based sample of older women. J Womens Health.

[39] Diaz AM, Kolber MJ, Patel CK, Pabian PS, Rothschild CE, Hanney WJ (2013). The efficacy of yoga as an intervention for chronic low back pain: a systematic review of randomized controlled trials. Am J Lifestyle Med.

[40] Field T (2016). Knee osteoarthritis pain in the elderly can be reduced by massage therapy, yoga and tai chi: a review. Complement Ther Clin Pract.

[41] Cramer H, Klose P, Brinkhaus B, Michalsen A, Dobos G (2017). Effects of yoga on chronic neck pain: a systematic review and meta-analysis. Clin Rehabil.

[42] Park J, Krause-Parello CA, Barnes CM (2020). A narrative review of movement-based mind-body interventions: effects of yoga, tai chi, and qigong for back pain patients. Holist Nurs Pract.

