# Supplementary material for: Feasibility of a yoga intervention to decrease pain in older women: a randomized controlled pilot study
Source: BMC Geriatr. 2020 Oct 12;20:400. doi: 10.1186/s12877-020-01818-y (PMC7552447; doi:10.1186/s12877-020-01818-y)
Supplement: Supplementary file 1 [file 12877_2020_1818_MOESM1_ESM.docx]

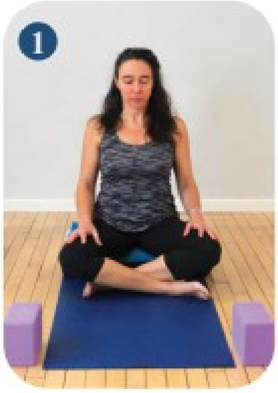

Three-Part Breath

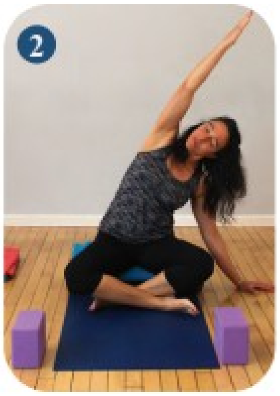

Seated Side Flow

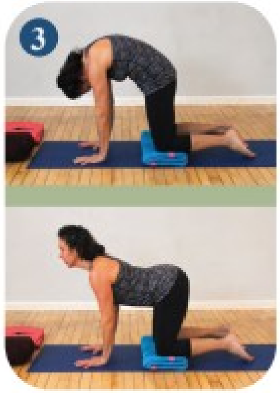

Cat/Dog

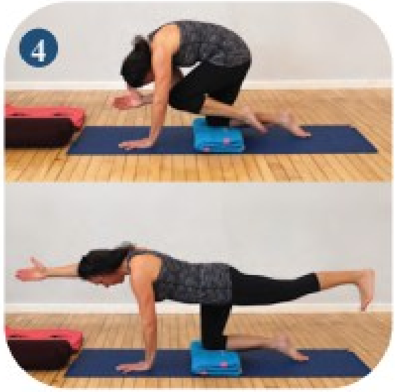

Leg Extension

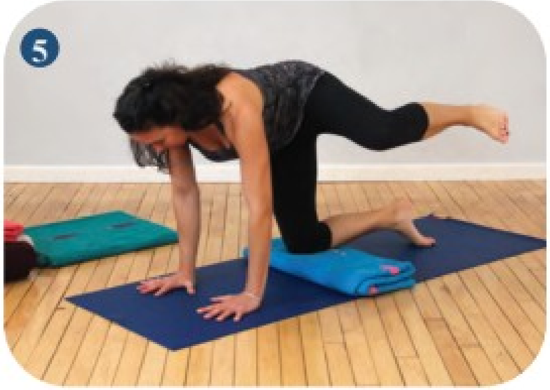

Hip Circles

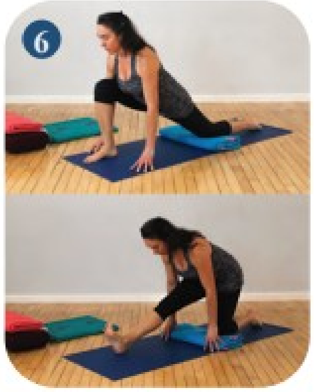

Rocking Flow

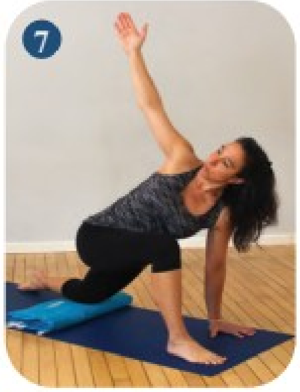

Lunge Twist Flow

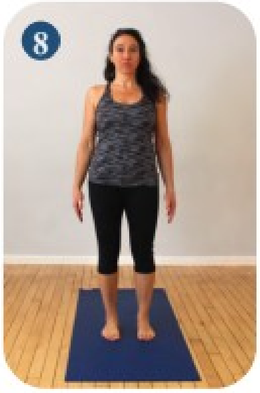

Mountain Pose

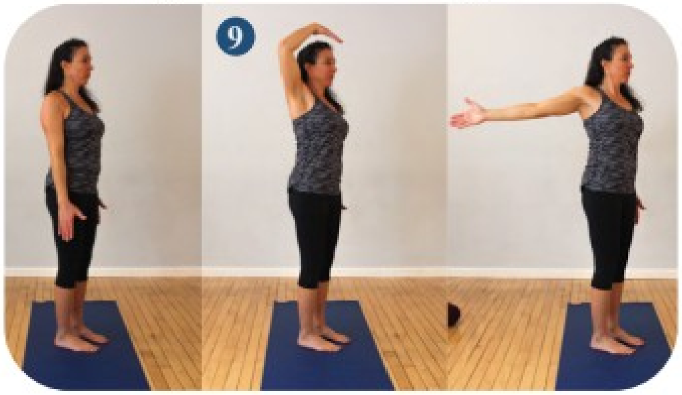

Greaser Arms

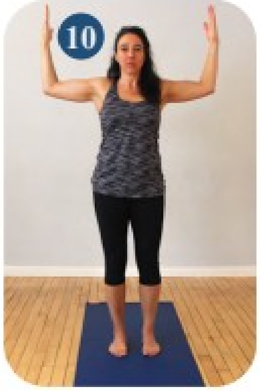

Bird Wings

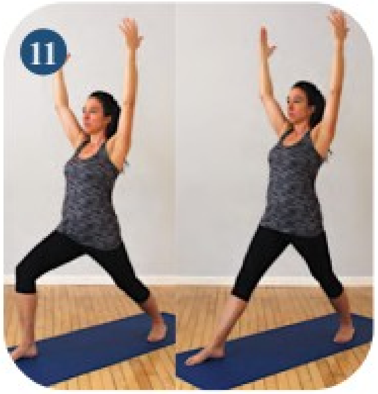

Warrior I

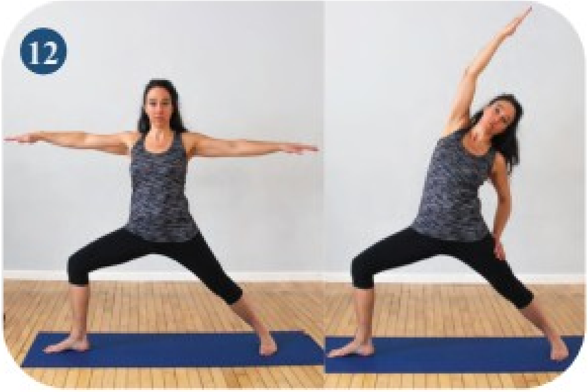

Warrior II to Peaceful Warrior

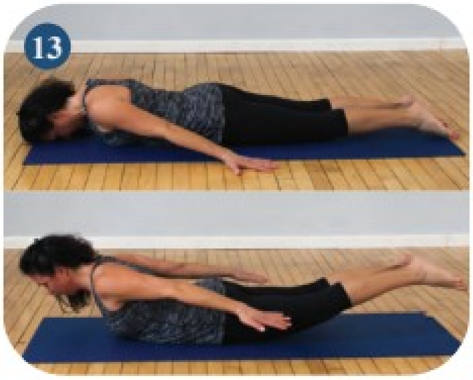

Boat Pulse

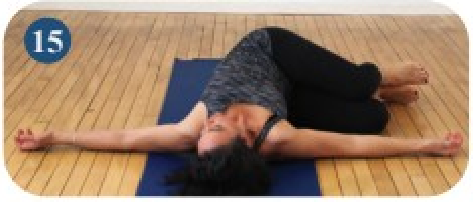

Supine Twist Flow

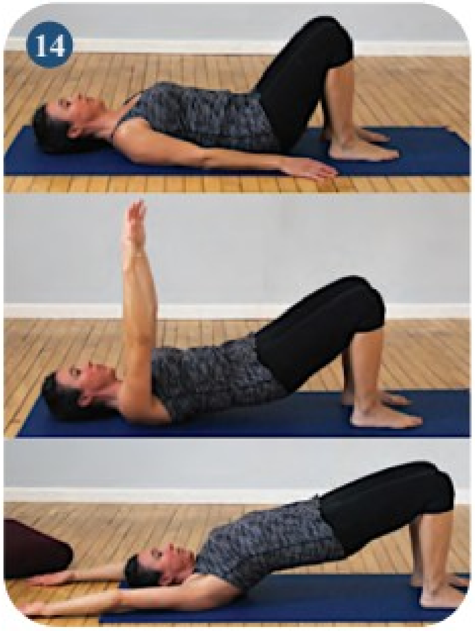

Bridge Kriya

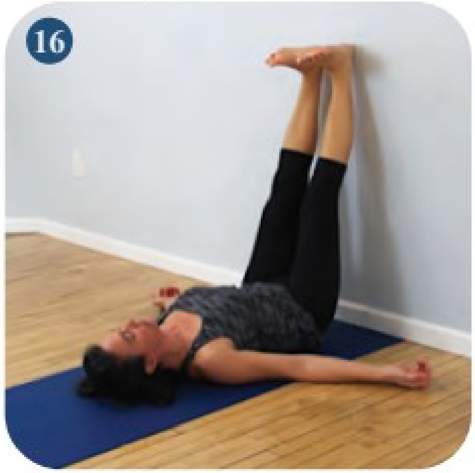

Legs Up the Wall

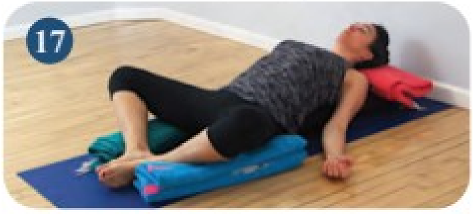

Supported Reclined Bound Angle

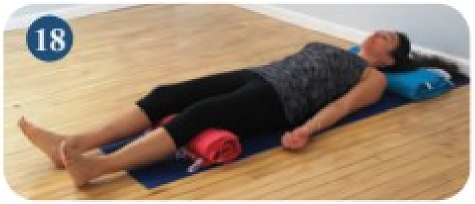

Savasana
